# Coalitions for Sustainable Finance and Sustainable Development

**DOI:** 10.1134/S1019331622080032

**Published:** 2022-06-29

**Authors:** Yu. A. Danilov

**Affiliations:** Russian Academy of National Economy and Public Administration (RANEPA) under the President of the Russian Federation, Moscow, Russia

**Keywords:** sustainable development, sustainable finance, ESG coalitions, responsible investment, ESG principles, impact economy, greenwashing

## Abstract

This article deals with the formation of coalitions for sustainable development and sustainable finance in developed countries and in Russia. In developed countries, broad national coalitions for sustainable development have been formed based on the initially established industry coalitions of investors and financial institutions for sustainable finance. The ideological core of such coalitions is the idea of new models of capitalism based on the principles of sustainable development as an ideal social structure. The concepts of stakeholder capitalism and the impact or caring economy are examples of such models. In Russia, similar coalitions are much narrower because of the imitation of following the environmental, social, and governance (ESG) principles and mass greenwashing. At the same time, there are objective factors that can lead to the expansion of ESG coalitions and strengthening incentives for the implementation of a sustainable development model in Russia.

## INTRODUCTION

Over the past 10–15 years, developed countries have been rapidly moving towards a new ideology of economic development, which places environmental and social values and goals at the forefront. It is referred to as sustainable development, which emphasizes the fact that economic agents following its guidelines are forced to balance different, and to a large extent, contrary goals. Since the traditional paradigm of economic sciences recognizes the simultaneous pursuit of different goals as irrational, the new ideology requires a radical transformation of theoretical ideas about the activities of economic agents, including modifying the concept of value and rejecting a number of fundamental concepts of the traditional paradigm. Perhaps, in addition to rigid, mathematically rigorous models, more flexible methods of evolutionary psychology, biotechnology, and cybernetics will be actively used. For more information on the attempted theoretical revolution related to the growth of responsible investment, which is one of the most important elements of sustainable development, see (Danilov, 2021b).

The transition to a sustainable development model is not limited to changes in the economy and economic science. Transformations are underway in a large part of humankind’s ideas of purposes and values, resulting in changes across the entire spectrum of human sciences and in the prevailing ideological imperatives.

The goals of sustainable development are set out in a United Nations document (United Nations, 2015). Many international organizations and states, guided by these goals, are developing plans for the transition to the new model. However, in recent years, the private sector has been more dynamically striving for sustainable development, largely under the influence of the activity of private investors, who voluntarily impose restrictions on the placement of investments. This type of investment is called responsible investment.

According to the UN-supported Principles for Responsible Investment (PRI) Initiative, the total value of assets under management of responsible institutional investors who subscribe to the PRI increased from $6.5 trillion in 2006 to $103.4 trillion in 2020,^1^ by almost 16 times.

The rapid growth of responsible investment and the subsequent introduction of ESG (environmental, social, and governance) standards in the functioning of financial markets have predetermined the special role of sustainable finance in the sustainable development model. It is through the financial markets that private capital has influenced the consolidation of the entrenchment of the new model of sustainable development as a consensus vision of a “beautiful future” of mankind. It should be noted that it was through this channel that the largest entrepreneurs in developed countries influenced public opinion by their example, investing their own capital in accordance with the ESG principles. The author did not find works that empirically confirm the importance of the responsible investment of individual private (family) capital or the capital of publicly significant individuals. However, the assumption that the public participation of the major entrepreneurs in responsible investments played the role of an incentive motive in the rapid increase in their volume seems reasonable (Danilov, 2021a).

There are two main blocks of reasons that caused an intensive movement towards sustainable development.

First, society’s awareness of sustainable development has increased as a response to mounting environmental problems. They are increasingly evident in the scarcity of resources, the aggravation of social problems, and the growing global interdependence of mankind (the advent of the “age of internalization”).

Second, there were additional factors in the financial sector. Responsible investors who want to contribute to solving global problems have emerged, and there is a demand for long-term confidence as a response to ultralow interest rates, cyclical risks, and financial instability (Danilov, 2021a).

The most prominent concerns have been about environmental risks, which is why for a long time (until 2020) “green” financial instruments developed faster than social ones. Green bond issuance typically accounted for more than 80% of total ESG-compliant bond issuance.

Experts at the World Economic Forum named four environmental risks among the five global risks with the highest probability of realization in 2021: extreme weather, losses from climate events, destruction of human habitats, and loss of biodiversity. In 2020, all five risks on the list were environmental; and in 2019, three out of five. Among the five strongest risks affecting the socioeconomic system in 2021, three were environmental (in 2020 also, three of the five were environmental, and in 2019, two were environmental) (WEF, 2021).

The coronavirus pandemic and the new social risks it has created have led to a more active issuance of social bonds. At the end of 2020, the volume of issues of social bonds and sustainable bonds (issued to finance environmental and social programs) for the first time equaled the volume of green bond issues (S&P Global Ratings, 2021).

Unlike developed countries and the largest developing economies, which are increasingly actively embarking on the path of sustainable development, Russia has so far remained extremely passive in this movement. In my opinion, this situation has developed due to the difference in the breadth of coalitions for sustainable development and sustainable finance in the world and in Russia.

## COALITIONS FOR SUSTAINABLE DEVELOPMENT AND SUSTAINABLE FINANCE IN DEVELOPED COUNTRIES

Broad coalitions for the introduction of the principles of sustainable development and sustainable finance in economic life were the most important factor that predetermined the accelerated development of sustainable finance and the idea of sustainable development in developed countries.

The “tragedy of the commons” is a classic problem in environmental economics. It describes a situation in a shared resource system where individual users, acting independently in accordance with their own interests, behave contrary to the common good of all users, depleting that resource through their collective actions. Common resources include not only natural resources that can be depleted but also, for example, air or water that can be overused by consumers. The common good is usually maintained through government taxation/regulation or the transfer of private property rights (Schoenmaker, 2017, p. 60).

E. Ostrom (Ostrom, 1990) proposed principles for the sustainable and equitable use of common resources. The main idea is to create coalitions that develop rules for the use of the common good, monitor the behavior of members, apply incremental sanctions against rule breakers, and provide affordable means to resolve disputes. To create an effective coalition, it is necessary to clearly define its boundaries in order to engage the most influential stakeholders as much as possible and to ensure that those affected by the rules can participate in changing them.

Following the principles of the design of coalitions developed by Ostrom (Ostrom, 1990), D. Schoenmaker considers the following features of coalitions for sustainable finance (Schoenmaker, 2017):

(1) well-defined boundaries of what percentage of the relevant group is covered by the coalition;

(2) membership rules that limit the use for the common good by local conditions. This can then be translated into the sustainable finance typology to which the coalition adheres;

(3) collective choice mechanisms: those affected by the rules and principles can participate in changing them;

(4) monitoring: reporting on compliance with the rules and principles, as well as an independent assessment of the degree of their compliance;

(5) sanctions and rewards: a system of penalties for participants’ violations of the rules and rewards for their compliance;

(6) conflict resolution mechanism: quick access to inexpensive mechanisms for resolving conflicts between members or between members and officials.

Schoenmaker proposes that the rules governing the use for the common good, such as an affordable carbon budget, should follow a systems approach. He gives examples of the most powerful industry coalitions (in the wealth management industry, in the banking sector, and in the corporate sector) for sustainable finance (Schoenmaker, 2017, pp. 62–63):

– asset managers who share the principles of responsible investment (PRI signatories);

– asset managers who in their investment strategies focus on creating global long-term value (Focusing Capital on the Long Term Global, FCLT Global);

– banks sharing the Equator Principles;^2^

– banks that are members of the Global Alliance for Banking on Values (GABV);

– corporations developing the principles of sustainable finance within the framework of the events and programs of the World Economic Forum (WEF);^3^

– corporations that are members of the World Business Council for Sustainable Development (WBCSD).

What encourages long-term investors to join new sustainable finance coalitions? One of the main incentives is access to opportunities to transition to a sustainable economy. Members of the PRI, FCLT Global, GABV, and WBCSD are inherently motivated to work towards long-term value creation. Other investors may be attracted to the argumentation of alliances of financial institutions that advocate sustainable finance, or they may be compelled to follow these principles by cooperation with corporations that share them and limit their business contacts on these grounds. Investors may be interested in joining in order to avoid the risk of losing assets. Collective investor advocacy by the coalition to encourage governments to clarify their agendas, for example in relation to climate change mitigation (including the timing of regulations and taxes), could reduce political uncertainty about the future value of assets.

Coalitions for sustainable development are much broader than coalitions for sustainable finance. As a rule, they are not formed according to the industry principle, since there were no economic incentives for entrepreneurs from “brown” industries to switch to green technologies before the emergence of a large group of responsible investors. The growth in the share of responsible investment, the activity of coalitions for sustainable finance, and the initiatives of individual entrepreneurs, investors, politicians, and scientists created such incentives and predetermined the formation of broad-based community-wide coalitions for sustainable development.

Such coalitions are formed after the idea of sustainable development captures the whole society. They are built to a large extent on the same principles as the sectoral (guild and industrial) associations for sustainable finance that arose earlier.

At the level of public policy in developed countries, both leading political forces and weaker parties and social movements are currently promoting sustainable development. In a number of countries, this idea is actively promoted by national governments.

National banks show a generally neutral attitude. As a rule, they are not actively involved in the agenda, but they are under significant pressure from political organizations and independent experts who demand that at least the green factor be taken into account in the ongoing monetary policy.

Among non-financial corporations, a significant proportion of companies that benefit from sustainable finance follow the principles of sustainable development. Financial intermediaries, consultants, and auditors are actively involved in setting the agenda on the topic.

The role of responsible investors continues to grow, their share in the total volume of investments is very noticeable, and in some countries it is prevalent. The shares of assets invested in accordance with the PRI and ESG principles in the total volume of professionally managed assets in 2020 were as follows: Canada 62%, the European Union (EU) 42%,^4^ Australia 38%, United States 33%, Japan 24% (The Global Sustainable Investment Alliance, 2021).

## MODELS OF CAPITALISM BASED ON THE PRINCIPLES OF SUSTAINABLE DEVELOPMENT AS THE IDEOLOGICAL CORE OF COALITIONS

The broadest possible coalitions are formed based on common ideas about the ideal social order. In the case of coalitions advocating the transition to sustainable development, these ideas proceed from the fact that the transformation of the goals of socioeconomic development in accordance with the goals of sustainable development leads to the emergence of a conceptually new model of capitalism.

At the same time, its formation is perceived as a defense of capitalism itself, understood as an exceptional value that made people rich and free. Development according to the Washington Consensus, a system of beliefs centered on the free market as the most important factor in growth, has now been declared a mistake, since the effectiveness of the capitalist system depends on a much larger number of institutions (Henderson, 2021, p. 228). In earlier works, the goal was to protect capitalism from capitalists (Zingales, Rajan, 2004). In contrast, supporters of sustainable development propose new versions of capitalism, using both the models formulated by L. Zingales and R. Rajan (an open economy opposed to the model of crony capitalism), and the division of institutions into inclusive and extractive, which was described by D. Acemoglu and D. Robinson (Acemoglu, Robinson, 2015). Obviously, the idea of a sustainable development model takes into account the positions of leftist ideologies to a greater extent than the model of an open (competitive, liberal) economy, which contributes to financial development in general (Danilov, 2020).

At the same time, supporters of sustainable development, first, are extremely strictly in favor of “a transparent democratic government, as well as other institutions of an open inclusive society, including the rule of law, universal respect for truth and independent media” (Henderson, 2021, p. 221), and, second, they clearly separate the models of sustainable development from the model of the state economy (Schoenmaker, 2020).

In my opinion, the following two concepts (models) of sustainable development, which are at the center of public discussions, are the most popular ones:

– the concept of stakeholder capitalism; and

– the concept of the impact (or caring) economy.

The concept of stakeholder capitalism is actively promoted by the head of the World Economic Forum K. Schwab. He defines it as a model for organizing a society in which private corporations take care of the public interest, which, in his opinion, corresponds best to the social and environmental challenges of our time (Schwab, 2019). Accordingly, the organization he leads is developing a conceptual framework for a new model of capitalism and compliance metrics.

The concept of a caring economy was developed by Schoenmaker. It is an economy in which governments and corporations balance profit on the one hand and the achievement of sustainable development goals on the other. The author subsequently renamed this conceptual model as the impact economy (Schoenmaker, 2020). The renaming eliminated the duality of terminology in describing both the concept of impact investment and the corresponding conceptual model of the economy. This concept relies to the greatest extent on the theoretical foundations, including the comparative analysis of D. Kopstein and M. Lichbach (Kopstein, Lichbach, 2005). It is much broader than the concept of stakeholder capitalism and uses the latter as one of its constituent elements.

Schoenmaker formulated the following main differences between the impact economy paradigm and the current paradigm of the economic system of capitalism (Table 1).

**Table 1.** The difference between the impact economy and the current economy

Parameter	Current paradigm	Impact economy
Goals		
At the level of the economy	Stimulating GDP growth	Well-being in a broad sense
At the corporate level	Profit maximization	Managed goals
Decision-making criteria		
At the level of the economy	Public benefit based on fiscal and economic indicators	Public benefit based on fiscal, economic, social, and environmental indicators
At the corporate level	Net present value based on financial factors (maximization of financial value)	Net present value based on integral cost^1^ (integrated value maximization)
Control		
At the level of the economy	Parliament	Parliament
At the corporate level	Shareholders	Stakeholders
Reporting		
At the level of the economy	Budget	Welfare budget
At the corporate level	Financial statements	Integrated reporting

Source: (Schoenmaker, 2020, p. 2)

^1^The concept of integrated value involves taking into account not only financial but also social and environmental aspects. It is also referred to in the literature as the total value, true value, etc.

The impact economy model is positioned as the golden mean between the market and state economy models.

In a market economy, the government is responsible for public goods (nonexclusive and noncompetitive goods) and creates the conditions for economic growth. Private companies produce and sell goods on the market without considering the social or environmental impacts. Companies operate in the interests of their shareholders. The common good is the exclusive province of government.

In a public economy, the government (the state) is powerful and responsible for the production of both public goods and, in large part, private goods. Companies can produce private goods, but they are largely owned by the state.

In his work, Schoenmaker (Schoenmaker, 2020) compares the quantitative characteristics and measures of the qualitative parameters of three countries, which are the most pronounced examples of the three types of modern economic systems: United States (market economic system), EU (impact (caring) system), and China (state system).

Europe is indeed an intermediate link between the market and state economies in terms of GDP per capita, level of competition, business dynamics, and receptivity to innovation. At the same time, in terms of social and environmental well-being, Europe is ahead of both the United States and China. Europe (compared to the United States and China) has the lowest level of inequality (according to the Gini coefficient), the highest level of human rights, the highest level of gender equality, the lowest level of carbon dioxide emissions, and the highest level of forest cover. As a result, Europe has the highest level of the SDG Index and the largest share of taxes in GDP. The European Union was one of the first in the world to embrace sustainable development (Ponedelko, 2021), which establishes it as a leader, together with New Zealand and Canada, in moving towards a sustainable development model.

Table 2 shows the values of the two main integral indices (SDG and CSR), which take into account the progress of countries in various areas of sustainable development for the three selected types of economic systems and Russia.

**Table 2.** The values of integral indices of sustainable development for three types of economic systems and for Russia

Rating	Economic system			
	market (United States)	impact (EU)	state (China)	Russia
SDG	73.0	78.1	70.1	68.9
CSR				
General	69.0	78.8	53.0	31.0
Social	65.2	83.5	52.0	28.0
Ecological	70.8	88.2	59.5	23.0

Source: (Schoenmaker, 2020); for Russia, calculations by RANEPA’s employees D.A. Pivovarov and I.S. Davydov.

Adding CSR to the analysis of ratings shows how far Russia is lagging behind in the field of sustainable development, at least in terms of the metrics that responsible investors take into account.

## COALITIONS FOR SUSTAINABLE DEVELOPMENT AND SUSTAINABLE FINANCE IN RUSSIA COMPARED TO COALITIONS IN DEVELOPED COUNTRIES

Obviously, without a broad coalition of interest groups focused on the development of sustainable finance in Russia, it is not possible to introduce the principles of sustainable finance in the Russian financial sector in the near future.

At the same time, the introduction of environmental protection goals in the practice of Russian corporations in Russia can receive quite strong support at the political level. The modern Russian political elite, in its activities, analyzes and takes into account the requests that are supported (or may be supported in the future) by a significant part of society. However, in practical politics, requests are taken into account, provided that they do not threaten the political elites.

It seems that the demands of society for environmental protection and for the intensification of socially oriented measures correspond to these conditions. It cannot be ruled out that these requests will be supported and implemented in political decisions.

At the same time, it should be taken into account that the green agenda is not included in the programs of parliamentary parties, and the slogans of sustainable development in terms of social problems are formulated to a large extent by the “non-systemic” opposition. These factors significantly reduce the power of the coalition in its political part.

However, we can assume the following composition of the coalition for sustainable finance in modern Russia:

– green political movements that exert soft political pressure;

– representatives of state power who seek to intercept the social agenda from the opposition;

– state corporations looking for an opportunity to apply their resources in various aspects, including in terms of the economic and public effect;^5^

– brown non-financial corporations, which are afraid of incurring significant losses related to the entry into force of the border corrective carbon tax of the EU;

– other non-financial corporations that see an additional resource in attracting investments in global markets through the interest of responsible investors and in the form of using the investment resources of state corporations;

– consultants and experts seeking to earn additional income on a fashionable topic;

– Moscow Exchange, actively involved in the sustainable finance agenda through participation in international organizations (primarily, the World Federation of Exchanges) and discussion clubs;

– Bank of Russia (subject to the intensification of efforts to promote the ideas of sustainable finance in Russia and their implementation in monetary policy).

The composition of a potential coalition differs significantly from similar associations in developed countries. However, even the list given above is only a potential composition of the coalition for sustainable finance in Russia, and the actual coalition is significantly narrower. In Table 3, the author presents his vision of the differences between coalitions for sustainable finance in Russia and in the world.

**Table 3.** Comparison of the composition of coalitions for sustainable finance in developed countries and in Russia

	In developed countries	In Russia
Public policy	Leading political forces represented in parliament	Predominantly politicians outside the parliament, incl. “non-systemic”
Government and quasi-government	National governments, in a number of countries very actively involved in the process	The active role of VEB.RF, as well as the more-or-less active role of other state corporations and individual federal executive bodies
National banks	Generally neutral attitude; lack of active participation	The Bank of Russia is becoming increasingly actively involved in the agenda, and in general, has a positive attitude
Non-financial corporations	A significant proportion of corporations benefiting from sustainable finance follow the ESG principles	Isolated cases of formal adherence to the ESG principles (all of them involve listing on a global exchange); the activities of many large corporations destroy the environment, which makes them opponents of the ESG
Financial intermediaries	Extremely active participation in the agenda	Generally neutral attitude; lack of active participation
Consultants and auditors	Extremely active participation in the agenda	Extremely active participation in the agenda
Investors	The role of responsible investors is growing rapidly, they are the main driving force behind ESG coalitions	Responsible investors do not exist as an independent group (there are no domestic investors; foreign investors consider Russia as a negative example in addition to sanctions)

Source: compiled by the author.

Financial intermediaries, by themselves, do not generally act as conduits for the concept of sustainable finance. They are actively involved in the implementation of the concept when they see a noticeable demand from investors and other categories of consumers of financial services. Therefore, it can be assumed that financial intermediaries in Russia may also be actively involved in the implementation of the concept of sustainable finance when they see demand for its elements from investors.

Some Russian corporations whose shares are traded on foreign exchanges became involved in promoting sustainable development standards in 2020, when the coronavirus pandemic forced global corporations to increase their attention on sustainable development issues, which was also reflected in the actions of global stock exchanges. For example, only in October 2020, MTS announced that it had “started preparing an updated strategy for sustainable development and corporate social responsibility until 2025.”^6^

Another part of Russian corporations, exporters of carbon-intensive products to the EU, took up the problem only in 2021, when it became clear that a carbon tax would be introduced in the very near future. Their response has been mixed: only a small proportion of these corporations have actually taken up the challenge of reducing their carbon footprint, while the majority intend only to imitate such activities, actually engaging in greenwashing—creating a deceptive image of a green company—by misleading investors.

Unlike other countries, in Russia the state is still extremely weakly involved in sustainable development and sustainable finance. At the federal level, the development of incentives for the development of instruments of sustainable finance in Russia is just beginning; however, so far this process concerns only green financing instruments.

In April 2019, the Russian government approved a resolution,^7^ according to which enterprises have the right to reimburse the costs of paying coupon income on bonds issued as part of the implementation of investment projects to introduce the best available technologies.

Decree of the Government of Russia No. 1912-r dated July 14, 2021 approved the “Goals and main directions of sustainable (including green) development of the Russian Federation.” The document very accurately characterizes the attitude of the government to the problems of sustainable development. It is a document of only 3 pages, containing both the goals and the main directions of sustainable development. Of course, the appearance of such an order, even this most concise order, is a small step towards civilization. However, out of all areas of sustainable development, the government has limited itself to green issues and has ignored social issues.

The draft law “On amendments to the Federal Law “On the electric power industry” and certain legislative acts of the Russian Federation in relation to the Introduction of green certificates” began to be developed in 2019; however, it was terminated for unspecified reasons in 2021.

## THE CONSEQUENCES OF THE ABSENCE OF A BROAD COALITION FOR SUSTAINABLE DEVELOPMENT AND THE OPPORTUNITIES FOR IMPLEMENTING A SUSTAINABLE DEVELOPMENT MODEL IN RUSSIA

Russia’s sustainable development agenda intensified in 2020–2021. However, so far this process has not led to the formation of a broad coalition for sustainable development. The main consequence of the absence of a broad coalition for sustainable development in Russia is the imitation of activities in this direction. Mimicking government imitation does not incur direct losses for investors, but corporate imitation of sustainability transition activities can lead to it because it misleads investors.

The big problem of Russian corporations has always been a certain imitation of corporate governance, the mimicking the corporate behavior of Russian corporations with classic examples of corporate behavior. I. Belikov considers the gap between the formal legal role of the boards of directors of Russian public companies and the practice of corporate governance in them to be “catastrophic” (Belikov, 2019, p. 486). The habit of imitation has also persisted in the field of sustainable finance, which is being introduced in the practice of corporate governance, the disclosure of adherence to which is required by international stock exchanges where shares of Russian corporations are traded. By imitating adherence to sustainable development standards, corporations are engaged in greenwashing.

Greenwashing is a fairly common phenomenon in the world, since there are objective reasons for its existence related to the difficulties in assessing the compliance of corporate behavior with the ESG principles (shift in ESG ratings samples by the size of corporations and industry structure; lack of a holistic methodology and uniform standards for assessing ESG factors; etc. (see Danilov, Pivovarov, Davydov, 2021)). As the issuance of social bonds and other sustainable instruments grows, so does the spread of “sustainability-washing” practices (S&P Global Ratings, 2021).

However, in Russia, almost everything that is labeled as sustainable finance turns out to be greenwashing on closer inspection.^8^

In the context of the weakness of coalitions for sustainable development, the effective implementation of ESG principles in Russia is difficult. However, it is clear that there are at least three factors that can seriously change the situation for the better.

First, the EU’s position to force foreign trade partners to comply with these standards. The analysis showed that these EU measures will have the strongest affect Russia (60% of whose imports to the EU are energy products), especially after 2030 (Leonard et al., 2021). In addition to the existing rules for listing on global exchanges, this forms an extremely powerful incentive for corporations to actually follow the ESG principles.

Second, a fairly wide layer of responsible private investors can be created in Russia, which can significantly strengthen the coalition for sustainable development. In relation to this, the Bank of Russia has a gigantic resource, which can introduce the carbon neutrality indicator into monetary policy. Moving from a market-neutral to a carbon-neutral Lombard list will provide a significant advantage to investors in green bonds, finally creating incentives for responsible investment.

Third, financial regulators have every opportunity to suppress greenwashing,^9^ which will increase the reliability of responsible investments.

## Footnotes

1 About the P.R.I. Principles for Responsible Investment. https://www.unpri.org/pri/about-the-pri.

2 The Equator Principles are a risk management system adopted by financial institutions to identify, assess, and manage environmental and social risks in projects, primarily designed to provide the minimum standard of due diligence and monitoring to support responsible risk decision-making. https://equator-principles.com/about/.

3 To date (2022), numerous such events and programs have been formed within the WEF. Examples include the initiatives of the Getting to Zero Coalition and Mobilizing Investment for Clean Energy in Emerging Economies.

4 It should be noted that in the EU the share of responsible investors peaked in 2014 (59%), but then it declined as a result of the stricter criteria used for classifying investors as responsible (which is also typical for some other countries), and also because of the introduction of mandatory regulation (for example, disclosure of non-financial reporting for all companies of a certain size).

5 It is worth highlighting the actions taken by VEB.RF to create the foundations for the functioning of markets for instruments of sustainable finance in Russia. VEB.RF has developed and approved guidelines for the development of investment activities in the field of green finance in Russia and, as an appendix to these recommendations, a taxonomy of green investments in Russia, i.e., two fundamental documents, without which the functioning of the market for private responsible investment in the field of the green economy would not be possible.

6 In an address to shareholders, distributed through the National Depository Center, MTS explained its decision as follows: “The global situation with the pandemic has resulted in adjustments in the attitude of many companies to corporate social responsibility, putting the latter at the forefront of strategic business development. Today, corporations are forced to look for new approaches to solve problems, to act together in order to give the fight against the crisis the required impetus in the implementation of sustainable development goals. In this situation, the ESG factors have become even more relevant, since they allow us to assess the importance of ongoing changes for the future development of the business.” https://cadocs.nsd.ru/20201030%20MTS.pdf.

7 In the work (Danilov, Pivovarov, Davydo, 2021), the authors give a number of egregious examples; here, we give only one. Even the compilers of sustainable development indices (Moscow Exchange and the Russian Union of Industrialists and Entrepreneurs) in Russia give priority to companies from brown industries, which make up 62% of the lists of these indices.

8 The author does not share the reviewer’s opinion that the main tools to fight greenwashing is the national green taxonomy and existence of verification procedures. However, taxonomy and verification are indispensable for the effective suppression of greenwashing by instruments of financial regulation and supervision.

9 Decree of the Government of the Russian Federation of April 30, 2019 N 541 “On approval of the Rules for granting subsidies from the federal budget to Russian organizations to reimburse the costs of paying coupon income on bonds issued as part of the implementation of investment projects to introduce the best available technologies.”
